# Non-invasive brain stimulation in frontotemporal dementia: syndrome-specific signals and priorities for future trials

**DOI:** 10.3389/fnagi.2026.1842455

**Published:** 2026-06-24

**Authors:** Jingshu Wang, Xiaoshu Fu, Jiangru Liu, Yu Yang

**Affiliations:** Department of Neurology, The First Hospital of Jilin University, Changchun, China

**Keywords:** frontotemporal dementia, non-invasive brain stimulation, photobiomodulation, prefrontal stimulation, primary progressive aphasia, trial design

## Abstract

Frontotemporal dementia is the second most common cause of young-onset dementia after Alzheimer’s disease and lacks disease-modifying treatment. This narrative review summarizes human studies of non-invasive brain stimulation, including repetitive transcranial magnetic stimulation and theta-burst stimulation, transcranial electrical stimulation, and transcranial photobiomodulation, in frontotemporal dementia and primary progressive aphasia. We review stimulation targets, protocols, outcomes, and safety, and organize the evidence by clinical subtype and modality. Current data remain preliminary, but recurrent signals support prefrontal and cerebellar repetitive transcranial magnetic stimulation/theta-burst stimulation and prefrontal or temporoparietal transcranial direct current stimulation, particularly in primary progressive aphasia. By contrast, controlled evidence in behavioral-variant frontotemporal dementia is limited and heterogeneous. Future trials should be sham-controlled, multicenter, and syndrome-stratified; combine stimulation with symptom-relevant cognitive or language therapy; and incorporate biomarker-informed targeting, target-engagement measures, and longer follow-up to determine durability and clinical relevance.

## Highlights

- Frontotemporal dementia has no approved disease-modifying therapy; NIBS is a circuit-based candidate adjunct for symptom management.
- Evidence is strongest for tDCS and rTMS/TBS in PPA; bvFTD remains under-studied with heterogeneous designs.
- Targets should align with syndrome-relevant networks and individual atrophy patterns; imaging-guided neuronavigation improves reproducibility.
- Pairing stimulation with symptom-relevant behavioral/language therapy may improve transfer and durability.
- Trial interpretability requires target-engagement readouts (EEG/MEG, fMRI/fNIRS, TMS-EEG, MRS, PET) alongside clinical outcomes.
- Harmonized sham methods, adverse-event reporting, and core outcome sets are needed for cross-study comparison and meta-analysis.

- Durability and maintenance (booster schedules; home-based protocols) should be tested with longer follow-up.
- Syndrome-stratified, multicenter RCTs with pre-registration and transparent reporting are essential before clinical translation.

## 1 Introduction

Frontotemporal dementia (FTD) is the second most common cause of young-onset dementia after Alzheimer’s disease (AD), with a crude incidence of approximately 2.28 per 100,000 person-years and a prevalence of approximately 9.17 per 100,000 (Onyike and Diehl-Schmid, 2013; Urso et al., 2025). It encompasses a heterogeneous group of clinical syndromes and their underlying pathologies. The core phenotype is progressive behavioral/personality changes and/or language impairment, occasionally accompanied by motor neuron disease or Parkinsonism (Sieben et al., 2012). Clinically, FTD frequently presents as behavioral-variant FTD (bvFTD) or as primary progressive aphasia (PPA), particularly the semantic dementia variant (svPPA) and non-fluent/agrammatic variant (nfvPPA). Neuropathologically, TAR DNA-binding protein 43 (TDP-43), tau, and fused in sarcoma (FUS) proteinopathies predominate, and approximately 40% of cases are familial and mostly linked to pathogenic variants of C9orf72, microtubule-associated protein tau (MAPT), and progranulin (GRN) (Antonioni et al., 2023).

Disease-modifying therapies are currently unavailable. Approved drugs are largely aimed at symptom management, with modest benefits and frequent adverse effects. This therapeutic gap has renewed interest in non-invasive brain stimulation (NIBS), including repetitive transcranial magnetic stimulation (rTMS), transcranial electrical stimulation (tES; transcranial direct/alternating current stimulation, tDCS/tACS), and transcranial photobiomodulation (tPBM), which have shown promise in several neuropsychiatric conditions (Lefaucheur et al., 2014; Geng et al., 2025). In FTD, evidence is still emerging and scattered across syndromes and stimulation protocols; however, early studies have suggested potential gains in executive and language outcomes in selected patients.

Against this background, a critical narrative synthesis is warranted, as the available clinical literature on NIBS in the FTD spectrum remains heterogeneous across syndromes, stimulation modalities, and outcome domains. To improve transparency, this review was informed by focused searches of PubMed/MEDLINE, Embase, and Web of Science from January 1990 to December 2025, supplemented by backward citation tracking, using terms related to FTD and PPA in combination with major NIBS modalities, including rTMS, theta-burst stimulation (TBS), tDCS, tACS, and tPBM. We excluded non-English articles, conference abstracts lacking sufficient methodological detail, and studies not involving FTD-spectrum populations or NIBS interventions. Titles, abstracts, and full texts were screened by two authors, with disagreements resolved by consensus. We prioritized peer-reviewed human interventional studies; case reports and small case series were included when higher-level evidence was unavailable, whereas preclinical and mechanistic studies were used only to provide biological context. Given the substantial heterogeneity in syndromic diagnoses, stimulation targets, dosing parameters, and outcome measures, quantitative pooling was not considered appropriate. Instead, we conducted a narrative synthesis and organized the evidence using a subtype-by-modality framework. As this article is intended as a narrative review rather than a formal systematic review, no PRISMA workflow, quantitative meta-analysis, or formal risk-of-bias assessment was undertaken. Accordingly, this review aims to balance mechanistic plausibility with a critical appraisal of the strengths and limitations of the available clinical evidence. We first summarize key neurobiological and network-level mechanisms relevant to FTD, noting that these remain largely hypothetical and are supported primarily by preclinical or indirect evidence rather than established clinical proof of target engagement. We then critically evaluate interventional studies across the major FTD phenotypes–bvFTD, nfvPPA, and svPPA–with particular attention to target selection, stimulation parameters, adjunct speech-language or cognitive therapies, and durability of treatment effects. Finally, we discuss practical considerations for safety monitoring and outline priorities for future trials, including image- and network-guided personalization, target-engagement biomarkers, clinically meaningful endpoints, and longer follow-up.

## 2 Mechanistic rationale, clinical evidence, and safety of NIBS in FTD

### 2.1 NIBS modalities, paradigms, and individualized targeting

Non-invasive brain stimulation includes several techniques that can alter cortical excitability and network communication. In FTD, syndrome- and stage-specific atrophy indicates that targeting and dose cannot be considered fixed. Herein, we outline the main modalities and proceed toward image- and network-guided localization.

#### 2.1.1 rTMS

Repetitive transcranial magnetic stimulation induces an intracortical electric field through electromagnetic induction, thereby modulating excitability and firing patterns (Siebner et al., 2022). The effects are not confined to the stimulated gyrus and can spread through connected circuits (Koch et al., 2024). This is important in FTD, where the symptoms reflect network-level dysfunction. The protocol used is paramount. High-frequency rTMS (≥5 Hz) is often used to increase excitability, whereas low-frequency rTMS (≤1 Hz) is commonly used to reduce excitability. The TBS technique applies patterned bursts. Continuous TBS (cTBS) is generally inhibitory [long-term depression (LTD)-like], whereas intermittent TBS (iTBS) is usually facilitatory [long-term potentiation (LTP)-like]; however, responses vary with baseline state and tissue integrity.

#### 2.1.2 tES

Transcranial electrical stimulation (tES) is typically delivered via tDCS or tACS. tDCS provides a weak, constant current that subtly shifts the resting membrane potential, thereby changing the probability of neuronal firing. The strongest and most reproducible behavioral effects are often observed when stimulation is combined with training or therapy. Although anodal and cathodal montages are typically linked to opposite effects on cortical excitability, the direction and magnitude of these effects depend on the montage and baseline physiology (Nitsche et al., 2008). tACS delivers oscillatory currents intended to entrain and modulate ongoing neural rhythms and to adjust the phase relationships within and between networks (Antal and Paulus, 2013). This aligns with reports of oscillatory abnormalities in FTD; however, it remains unclear as to how often rhythm modulation translates into clinically meaningful and durable changes.

#### 2.1.3 tPBM

Transcranial photobiomodulation uses low-irradiance red to near-infrared light (typically 600–1,100 nm). The leading hypothesis is that this non-thermal mechanism involves chromophore absorption and mitochondrial/redox signaling. Downstream effects reported in experimental studies include increased adenosine triphosphate (ATP) availability, modulation of reactive oxygen species (ROS)/nitric oxide (NO) signaling, anti-inflammatory shifts, and neuroprotective signaling. These pathways overlap with the processes implicated in neurodegeneration, making tPBM biologically plausible, although FTD-specific dosing and targeting data remain limited (Nairuz et al., 2024).

### 2.2 Individualized targets and parameter optimization

The dorsolateral prefrontal cortex (DLPFC) is frequently selected because it is accessible and functionally broad, linking executive control with behavioral regulation, language, and social cognition. Cerebellar targets have attracted considerable attention (Antonioni et al., 2025). However, FTD syndromes differ in network vulnerability, and it is unlikely that a single cortical target will serve all patients equally. Targeting is being increasingly supported by imaging. Functional magnetic resonance imaging (fMRI) guidance (Jung et al., 2024) and neuronavigation (Song et al., 2022) improve localization, and network-based protocols aim at symptom-relevant nodes [e.g., the salience network (SN) and default mode network (DMN)]. Similar approaches have been tested for AD (Jung et al., 2024) and Parkinson’s disease (Ayache et al., 2024). In FTD, the rationale is practical: atrophy and disconnection can reduce the effective engagement of the intended circuit.

Most FTD protocols are short (20–40 min per session; daily or on alternate days; for 2–4 weeks; often totaling 10–20 sessions). Longer courses are feasible: a 52-week stimulation has been studied in AD; this motivates maintenance testing for FTD (Koch et al., 2025). tPBM is often delivered in longer home-based courses (typically 20–30 min per session) for weeks or months, reflecting its portability and favorable tolerability profile. However, specific systematic data on FTD are sparse, and the optimal wavelengths, sites, and doses remain unclear.

Finally, the baseline state is important. The connectivity and neurochemical milieu influence the physiological responses to stimulation (Vidal-Piñeiro et al., 2015; Hawco et al., 2018; Jung et al., 2022). Therefore, biomarker-informed targeting and dose selection may improve the reproducibility of future FTD trials.

### 2.3 Pathophysiology of FTD: key molecular and circuit-level processes

Frontotemporal dementia reflects the converging pathways from genetic risk to proteinopathy and selective circuit degeneration. The commonest pathogenic variants are C9orf72, GRN, and MAPT. These factors promote the dysfunction of disease-associated proteins, most often TDP-43 and tau, and seldomly FUS, leading to a predictable set of downstream problems, including synaptic failure, axonal transport disruption, glial activation, and progressive network disconnection (Bang et al., 2015).

#### 2.3.1 TDP-43 proteinopathy

TAR DNA-binding protein 43 pathology is characterized by cytoplasmic mislocalization and aggregation, with concomitant nuclear depletion, leading to widespread disturbances in RNA processing, neuronal homeostasis, and synaptic maintenance (Kim et al., 2010; Gao J. et al., 2025). Experimental and translational studies have linked TDP-43 dysfunction to excitatory synapse loss, impaired plasticity-related signaling, and progressive weakening of vulnerable large-scale brain networks (Brettschneider et al., 2015; Seeley, 2017; Ni et al., 2023). Emerging evidence further suggests that inhibitory interneuron vulnerability, together with sustained astrocytic and microglial activation, may contribute to excitation–inhibition (E/I) imbalance and circuit instability in FTD (Zhao et al., 2015; Bright et al., 2021; Thapa et al., 2025). From a translational perspective, these observations provide a biological rationale for interventions aimed at modulating cortical plasticity and network communication. However, direct evidence linking TDP-43–related pathology to responsiveness to NIBS in FTD remains limited.

#### 2.3.2 Tau pathology

Tau pathology contributes to cytoskeletal instability, impaired axonal transport, synaptic dysfunction, mitochondrial stress, and progressive network degeneration in FTD (Rawat et al., 2022). Although toxic gain-of-function and loss-of-function mechanisms have been proposed, the key translational implication is that tau-related disease disrupts neuronal communication and plasticity at synaptic and systems levels (Derkach et al., 2007; Ahmed et al., 2014; McInnes et al., 2018). Tau pathology also interacts with neuroinflammatory processes and may propagate across structurally and functionally connected regions, consistent with the network-based distribution of FTD syndromes (Vogels et al., 2019; Chen and Yu, 2023; Schoonhoven et al., 2023; Basheer et al., 2024). In GRN-related disease, impaired lysosomal and autophagic function may further promote tau accumulation (Lesport et al., 2024; Pollack et al., 2024). Collectively, these observations provide a plausible biological substrate for neuromodulatory approaches aimed at stabilizing vulnerable networks or restoring plasticity; however, they do not establish tau-related mechanisms as determinants of clinical response to NIBS in FTD.

### 2.4 How NIBS could act in FTD: mechanistic hypotheses aligned to clinical phenotypes

**FIGURE 1:**
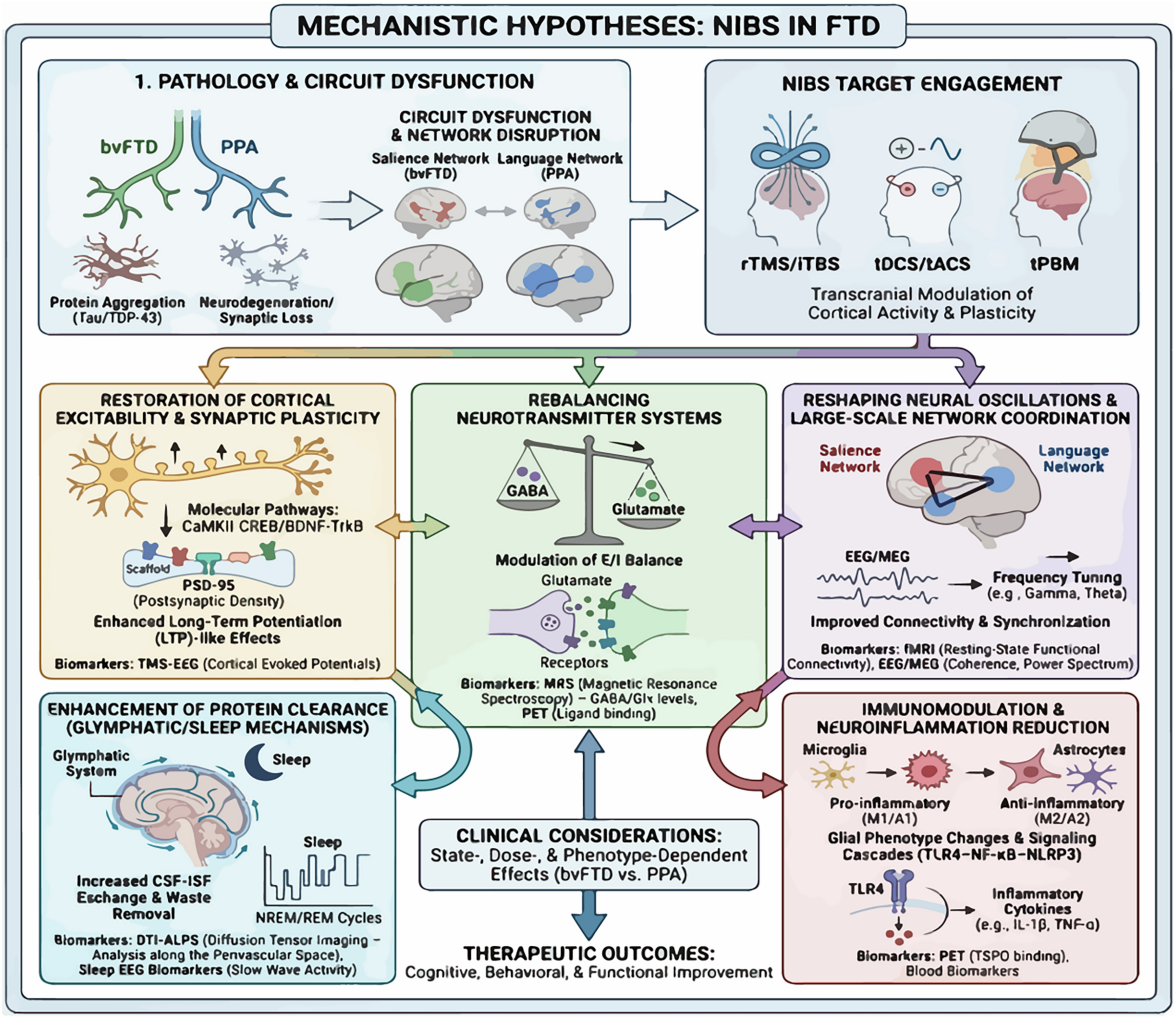
Mechanistic hypotheses for non-invasive brain stimulation (NIBS) in frontotemporal dementia (FTD) aligned to clinical phenotypes. The Figure summarizes proposed therapeutic mechanisms of rTMS/iTBS, tES (tDCS/tACS), and tPBM using a pathology → circuit dysfunction → target-engagement framework. Mechanisms are grouped into five overlapping domains: (1) restoration of cortical excitability and synaptic plasticity (e.g., CaMKII–CREB/BDNF–TrkB; synaptic markers such as PSD-95); (2) correction of excitation–inhibition imbalance, indexed by SICI/ICF and MRS Glx/GABA; (3) modulation of oscillations and large-scale network coordination (salience and language networks) measurable with EEG/MEG and imaging (fMRI/fNIRS); (4) immunomodulatory and anti-inflammatory effects (e.g., TLR4–NF-κB–NLRP3) with antioxidant responses; and (5) enhancement of protein clearance pathways (glymphatic/meningeal lymphatic), potentially linked to sleep and assessable with diffusion proxies (e.g., DTI-ALPS) and sleep measures. Candidate target-engagement biomarkers include TMS–EEG indices, MRS metabolites, synaptic PET, network imaging metrics, inflammatory markers, and glymphatic/sleep readouts. Effects are expected to be state-, dose-, and target-integrity dependent across bvFTD and PPA variants.

A mechanistic account of NIBS in FTD requires integration across molecular pathology, circuit dysfunction, and measurable target engagement. Within this framework, current hypotheses can be organized into five broad domains: synaptic plasticity, neurotransmitter balance, oscillatory and network coordination, neuroinflammation, and protein clearance pathways. However, the evidence supporting these mechanisms in FTD remains limited, with most data derived from preclinical studies, biomarker research, or NIBS investigations in other neurological and psychiatric disorders. Accordingly, these mechanisms should be viewed as plausible and testable frameworks that may guide future research, rather than as confirmed explanations of therapeutic effects. The proposed framework is summarized in Figure 1. The following sections are therefore intended to contextualize potential therapeutic hypotheses rather than to imply established, disease-specific mechanisms of clinical benefit.

#### 2.4.1 Restoring excitability and synaptic plasticity

Synaptic loss and impaired plasticity appear early in FTD and may precede or exceed structural atrophy (Brun, 1987). In bvFTD, synaptic PET with [^11^C]UCB-J has shown widespread synaptic density reductions that correlate with cognitive impairment and are disproportionate to gray matter loss (Malpetti et al., 2023). Complementary evidence from tau models points to selective vulnerability in SN regions with the depletion of postsynaptic density protein 95 (PSD-95), reduced postsynaptic density, and impaired AMPA receptor (AMPAR)/NMDA receptor (NMDAR) function (Warmus et al., 2014). Human tissue studies have also reported reduced synaptic protein density (Clare et al., 2010). Neurophysiology suggests that plasticity changes may begin before symptoms. Carriers of asymptomatic mutations show reduced LTP-like cortical plasticity, and symptomatic carriers show reduced short-interval intracortical inhibition (SICI) (Benussi et al., 2016). Therefore, NIBS is often framed as a method for biasing activity-dependent plasticity. rTMS and iTBS can induce LTP-like physiological effects and modulate network connectivity (Siebner et al., 2022). In dementia models, tPBM can improve hippocampal LTP/LTD and paired pulse measures, with behavioral gains (Buendía et al., 2022).

Across stimulation modalities, experimental evidence suggests convergent effects on synaptic plasticity and neurotrophic signaling, including pathways involving brain-derived neurotrophic factor and related plasticity-associated proteins (Mizuno et al., 2003; Podda et al., 2016; Ma et al., 2017; Heo et al., 2019; Yu et al., 2019; Thomson et al., 2020; Wu L. et al., 2022; de Oliveira et al., 2024; Nairuz et al., 2024; Sharbafshaaer et al., 2024; Chen et al., 2025; Toader et al., 2025). However, in FTD, these observations remain largely indirect and have not been consistently linked to clinical response. Accordingly, the practical implication is not that specific molecular cascades are established mediators of therapeutic benefit, but rather that future trials should integrate clinical outcomes with objective measures of plasticity and target engagement, where feasible, such as TMS–EEG indices, magnetic resonance spectroscopy, or synaptic positron emission tomography.

#### 2.4.2 Rebalancing neurotransmitter systems

Multiple lines of evidence suggest an E/I imbalance in the FTD spectrum. The “glutamatergic hypothesis” emphasizes abnormalities in AMPAR/NMDAR, synaptic release, and glutamate (Glu)-related excitotoxicity, potentially involving immune-mediated mechanisms (Benussi et al., 2019). MRS studies have reported reduced GABA and Glu levels in the right inferior frontal gyrus (IFG), with lower levels linked to behavioral disinhibition (Murley et al., 2020). Benussi et al. (2020b) have further reported that GABAergic/glutamatergic imbalance correlates with reduced “fluidity” of whole-brain dynamic functional connectivity, providing a plausible bridge from cellular E/I imbalance to network-level instability. In addition to Glu and GABA, dopaminergic and serotonergic abnormalities have also been implicated, and receptor metabolism co-localization analyses suggest subtype-specific vulnerabilities (Murley and Rowe, 2018; Premi et al., 2023; Bi et al., 2025). In principle, NIBS can be used to shift these systems. rTMS has been linked to changes in inhibitory interneuron function and GABA-related markers and to effects on neuronal survival and dendritic growth (Wang et al., 2025). In depression, high-frequency rTMS over the left DLPFC increases the combined signal of Glu and glutamine (Gln) Glx locally and contralaterally (Godfrey et al., 2021). In contrast, inhibitory 1 Hz rTMS to the motor cortex increased ipsilateral GABA and altered connectivity, consistent with an increase in inhibitory tone that stabilizes network activity (Gröhn et al., 2019). In preclinical studies, iTBS has been reported to alter extracellular GABA and Glu levels in hippocampal circuits and improve memory (Wu X. et al., 2024). The effects of tDCS on Glu and GABA are montage- and state-dependent (Harris et al., 2019). Regarding FTD, a double-blind randomized trial reported that 2 weeks of anodal tDCS over the left prefrontal cortex restored SICI and intracortical facilitation (ICF), consistent with the partial rebalancing of intracortical inhibition and excitation (Benussi et al., 2020a). Furthermore, dose-, target integrity-, baseline state matter-, and sex-dependent differences in the prefrontal Glx responses have been described (Dwyer et al., 2018; Vural et al., 2024). tPBM may indirectly influence neurotransmitter balance through bioenergetics, glial function, and inflammation. In depression models, PBM upregulates Glu transporter-1 (GLT-1), reduces extracellular Glu levels, rescues astrocyte loss, and increases AMPAR expression, thereby mitigating excitotoxicity (Zhang et al., 2021). Changes in 5-HT and NO levels have also been reported along with behavioral improvements (Eshaghi et al., 2019). Reviews have further suggested that PBM modulates ion channels, including Glu receptors (Zhang et al., 2024). Overall, neurotransmitter modulation remains a plausible but state-dependent pathway, and FTD trials will likely require baseline biomarkers to interpret variability. However, in FTD, these mechanistic inferences remain indirect, and their relationship to clinical response has yet to be established.

#### 2.4.3 Reshaping oscillations and large-scale networks

Frontotemporal dementia is associated with altered oscillatory dynamics and disrupted network topology. In bvFTD, MEG Go/No-Go studies have suggested reduced beta desynchronization within the right IFG (preSMA) motor network, with altered cross-frequency coupling related to impulsivity and behavioral disinhibition (Hughes et al., 2018). EEG graph analyses have reported reduced hub centrality and clustering, especially in frontotemporal systems, and a shift toward a stochastic network organization (Wu X. et al., 2024). Resting-state fMRI showed early SN changes in asymptomatic MAPT carriers, with structural damage emerging later, consistent with a connectivity-to-degeneration cascade compatible with axonal spread mechanisms (Bouzigues et al., 2025a). Presymptomatic C9orf72 carriers also show prominent SN deficits (Lee et al., 2017). In established bvFTD, SN connectivity reduction is common, whereas DMN findings are mixed (Ferreira et al., 2022). In PPA, network disruption differs by subtype, with stronger sensorimotor network changes in nfvPPA and more limbic network involvement in svPPA. Therefore, the relationship between atrophy and connectivity is complex (Bouzigues et al., 2025b). These observations have encouraged two design choices: network-informed targeting and network-level endpoints. High-frequency TMS over the DLPFC has network-specific effects on metabolism and connectivity, and metabolic changes can be used to track connectivity modulation (Eldaief et al., 2023). In AD, rTMS targeting the bilateral angular gyri increases SN connectivity (Wang et al., 2024). In bvFTD, tDCS has been reported to increase SN connectivity, although similar modulation is intermittently observed in healthy adults after tACS (Mansouri et al., 2019). In dementia populations, tACS can entrain rhythms in frequency-selective ways (theta, beta, and gamma), and phase-resetting effects may persist after stimulation (Bréchet et al., 2021). These findings make oscillatory targets mechanistically attractive in FTD; however, their link to durable clinical benefits remains to be demonstrated. tPBM may also modulate oscillations and network metrics. Prefrontal 1,064 nm tPBM increased alpha/beta power at rest, with spatial patterns distinct from those in thermal stimulation (Wang et al., 2021). Dementia case reports have described reduced delta/theta power, increased alpha/beta power, and connectivity, alongside cognitive improvements (Vrankic et al., 2022). EEG graph analyses suggest that even a single session can shift power topology and network metrics, correlating with working memory changes (Shahdadian et al., 2022). A functional near-infrared spectroscopy (fNIRS) study reported increased resting-state connectivity and improved efficiency metrics after prefrontal tPBM (Urquhart et al., 2020).

Collectively, these findings support the possibility that NIBS improves symptoms by restoring network coordination, particularly within the SN and language networks, through modulations of oscillations, connectivity, and topology. Therefore, trials should incorporate network-level target engagement measures (EEG/MEG, fMRI, and fNIRS) along with clinical outcomes. However, in FTD, these mechanistic inferences remain indirect, and their relationship to clinical response has yet to be established.

#### 2.4.4 Immunomodulation and neuroinflammation

Neuroinflammation is increasingly recognized as a component of the FTD disease process, particularly in genetic forms associated with altered microglial and astrocytic function (Comi and Tondo, 2017; Stephenson et al., 2018; Bright et al., 2019; Hashimoto et al., 2022). Inflammatory mediators can influence synaptic integrity and circuit stability; accordingly, immunomodulation has been proposed as a potential downstream pathway through which NIBS might exert therapeutic effects (Boulanger, 2009; Krsek et al., 2024). Genetic FTD provides biologically plausible links between disease mechanisms and immune dysregulation. For example, GRN and C9orf72 mutations have been associated with impaired microglial homeostasis and phagocytosis, pro-inflammatory glial states, and excessive complement activation, which may promote aberrant synaptic pruning and network toxicity (Hashimoto et al., 2022). Across stimulation modalities, preclinical studies suggest that rTMS, iTBS, tDCS, and tPBM can modulate inflammatory signaling and glial states (Cardoso et al., 2022; Walter et al., 2022; Zuo et al., 2022; Shen et al., 2024; Liu et al., 2025; Zhang et al., 2025). However, in FTD, these effects remain largely inferential rather than clinically demonstrated. Accordingly, neuroinflammation should be regarded as a candidate mechanistic domain for future translational investigation rather than an established mediator of therapeutic response.

#### 2.4.5 Protein clearance and glymphatic pathways

Clearance-related mechanisms, including glymphatic and lymphatic pathways, have been implicated in neurodegenerative disorders characterized by tau and TDP-43 pathology (Nedergaard and Goldman, 2020; Ishida et al., 2022). The glymphatic system contributes to the removal of misfolded proteins and is influenced by aquaporin-4 polarization and sleep-related physiological states (Nedergaard and Goldman, 2020; Ishida et al., 2022). In FTD, diffusion-based imaging studies, including ALPS approaches, suggest that impaired glymphatic function may be associated with pathological burden and disease progression (Jiang et al., 2023; Premi et al., 2024). Experimental evidence further indicates that NIBS may influence clearance-related processes, either directly or indirectly, through modulation of sleep-related mechanisms (Salehpour et al., 2022; Wu C. et al., 2022; Li et al., 2023; Oxana et al., 2023; Wu et al., 2023; Wang et al., 2024; Gao D. et al., 2025). However, evidence linking these pathways to therapeutic effects in FTD remains highly preliminary and is derived largely from non-FTD or preclinical studies. Accordingly, clearance mechanisms should be considered exploratory and hypothesis-generating rather than established explanatory frameworks for the currently limited clinical literature in FTD.

### 2.5 Clinical evidence and safety by syndrome

Across syndromes, current clinical evidence is constrained by small sample sizes, heterogeneous protocols, and limited use of sham controls. For interpretive purposes, this heterogeneity can be grouped into four main domains: clinical phenotype and disease stage; target selection and regional atrophy burden; stimulation parameters and cumulative dose; and outcome definitions, including whether stimulation is paired with speech-language or cognitive therapy. These domains likely interact. For example, the same stimulation may engage different neural circuits depending on regional atrophy, and short, single-session studies may underestimate effects that require repeated stimulation combined with task-specific training. Accordingly, the literature is most informative when interpreted in a syndrome-specific manner, with attention to alignment between stimulation targets and vulnerable networks, the use of adjunct behavioral therapy, and the durability and generalizability of observed effects.

#### 2.5.1 bvFTD

Clinical evidence for NIBS in bvFTD remains limited and heterogeneous, in targeting and outcomes. Reported benefits, where present, tend to involve neuropsychiatric symptoms (e.g., apathy and disinhibition); social cognition (e.g., theory of mind) (Cotelli et al., 2018); and executive functions, rather than global cognitive measures. TMS approaches, most commonly rTMS and TBS, have largely been explored in small sample sizes and frequently without robust sham control. In a single-arm 10-day study, 11 patients with FTD (including bvFTD) received 10 Hz rTMS over the bilateral DLPFC and showed improvements in cognitive performance and daytime functioning. Interpretation is constrained by the absence of a control condition, and larger sham-controlled trials are required (Antczak et al., 2018). tES, including tDCS and the emerging tACS, has produced mixed findings. This variability likely reflects dependence on network integrity, degree of atrophy, and baseline symptom profiles. Anodal tDCS over the left prefrontal cortex improved executive function, emotion recognition, neuropsychiatric symptoms, and neuropsychological measures in presymptomatic and symptomatic carriers of pathogenic GRN variants (Benussi et al., 2020a). In bvFTD, tDCS has been associated with improved accuracy and selectivity in understanding communicative intention (Cotelli et al., 2018). Case reports have described improvements in apathy-predominant behavioral disturbances and socio-occupational function after short tDCS courses, with benefits persisting at longer follow-up in certain individuals (Agarwal et al., 2016). Longer-term programs combining tDCS with cognitive training have been reported to slow decline and improve cognition, language, and daily function, although the evidence base remains limited (Tippett et al., 2024). Further research indicates that tDCS can significantly reduce the total Neuropsychiatric Inventory (NPI) score, with the effect lasting up to 4 weeks, accompanied by EEG-related power spectral changes (Ferrucci et al., 2018). Not all controlled studies showed positive results. One trial on bvFTD reported no significant changes in verbal fluency or NPI scores after stimulation (Huey et al., 2007). A double-blind, sham-controlled, crossover study found no language benefit after a single prefrontal tDCS session, and modeling suggested that cortical atrophy may reduce the effective electric-field strength (Sanches et al., 2022). Considered together, these negative findings suggest that variability in outcome may reflect multiple interacting factors, including disease stage and regional atrophy burden, stimulation dose and session number, target selection, and alignment between the chosen endpoint and the predominant clinical deficits of bvFTD, rather than simple inefficacy of NIBS. This issue is particularly relevant in bvFTD, in which salience network dysfunction is central to the syndrome and may not be adequately addressed by generic prefrontal stimulation alone. Consistent with this view, a network-guided comparison of anodal salience network stimulation and cathodal default mode network stimulation demonstrated differential effects on cognitive and behavioral measures, although the sample size was small and physiological findings require replication in larger cohorts (Pini et al., 2022). Taken together, these findings support a shift from purely anatomical targeting toward syndrome- and network-informed approaches. Future bvFTD trials should therefore predefine behaviorally meaningful primary outcomes, stratify patients by disease severity and atrophy burden, and evaluate dose optimization and target personalization rather than assuming that a single frontal protocol is appropriate across cohorts. Direct evidence for tPBM in bvFTD remains limited, and it should therefore be regarded as experimental in this population.

#### 2.5.2 PPA: nfvPPA and svPPA

Compared with bvFTD, the NIBS literature is larger in PPA and more closely aligned with symptom-driven targets within language networks. However, treatment response varies not only by PPA subtype, but also by disease stage, regional atrophy burden, stimulation protocol, and the choice of language outcome measures. Across studies, the clearest benefits are reported when stimulation is combined with structured speech and language therapy (SLT), consistent with a facilitative or “priming” role. In rTMS studies, a single-session protocol over the left DLPFC improved action naming and was associated with broader cognitive gains (Margolis et al., 2019). Individualized rTMS targeting language-related regions (e.g., the left anterior temporal cortex) has resulted in improvements in spontaneous speech, object naming, and reading, accompanied by metabolic changes interpreted as increased synaptic activity (Pytel et al., 2021). Subtype effects have been reported. After single-session stimulation, action naming improved in nfvPPA, whereas the svPPA group showed little change (Cotelli et al., 2012). Long-controlled protocols provide stronger signals. A 4-week rTMS course improved the Boston Naming Test and Western Aphasia Battery scores, with effects observed at 6 months (Huang et al., 2023). In another study, iTBS over the left DLPFC combined with language therapy for 6 months was associated with slower regional metabolic decline and improvements in language, functional independence, and neuropsychiatric symptoms (Fernández-Romero et al., 2025). These findings highlight the importance of session number, cumulative dose, and concurrent behavioral intervention. tES has often been studied as an adjunct to SLT, typically targeting the left IFG or other language hubs. Many single-site trials have reported that anodal tDCS enhances therapy-related language gains with some generalization and persistence (Finocchiaro et al., 2006; Wang et al., 2013; Cotelli et al., 2014; Tsapkini et al., 2014; Gervits et al., 2016). A recent 10-day iTBS treatment study measured improvements in language function across weaker areas in language testing, along with enhanced functional connectivity in language networks of all subtypes and other cognitive function-related neural networks (Paranhos et al., 2025). High-definition tDCS (HD-tDCS) targeting the left IFG led to significant improvement in patient letter fluency and was accompanied by changes in event-related potential (Dugas et al., 2025). However, these effects appear to be less consistent in svPPA. Roncero et al. (2019) reported that anodal tDCS augmented language therapy but produced minimal improvement in untrained item naming in svPPA. A randomized trial combining anodal tDCS over the left IFG with written naming/spelling therapy reported stronger and more durable benefits than sham therapy overall, but no clear advantage in the semantic variant (Tsapkini et al., 2018). Multifocal tDCS does not consistently outperform SLT alone (Borrego-Écija et al., 2023). A recent meta-analysis reported no significant effect on composite language outcomes, highlighting the heterogeneity of protocols, targets, and subtype compositions (Lomi et al., 2025). These attenuated or null findings are informative, suggesting that short treatment duration, insufficient dosing, suboptimal target selection, or overly broad outcome measures may obscure treatment effects in specific subgroups. Modifiers of responses include baseline cognitive/language performance (McConathey et al., 2017; de Aguiar et al., 2020b), regional atrophy (de Aguiar et al., 2020a), and sex- and age-related differences in network connectivity changes (Licata et al., 2023). Collectively, these factors indicate that variability across studies likely reflects interactions among subtype, disease burden, target engagement, stimulation parameters, and endpoint selection. Together, these findings support a move toward subtype-specific protocols and biomarker-informed stratification (atrophy patterns and network connectivity) with streamlined parameter optimization. Across subtypes, the most reproducible tDCS signal in nfvPPA comes from left IFG stimulation, combined with lexical retrieval or spelling/naming therapy; the benefits are typically strongest for trained items and may be generalized. In svPPA, the added benefit is less consistent, possibly reflecting more widespread semantic network degeneration and disconnection, and suggesting a need for network-based or individualized targeting and stricter stratification. Future studies should therefore stratify patients by subtype, atrophy pattern, and baseline language profile, and prioritize outcome measures aligned with the trained domain and the expected mechanism of benefit. However, direct clinical evidence for tPBM in patients with PPA remains scarce. Although PBM has been explored in other dementia or cognitive impairment cohorts and a few meta-analyses suggest short-term cognitive signals, its relevance to subtype-specific language outcomes in PPA remains to be tested.

### 2.6 Safety and tolerability

In small studies, NIBS was well-tolerated by patients with FTD and PPA. However, the reporting of adverse events (AEs) was uneven. Most trials were brief and too small to detect rare complications. Safety assessment was also syndrome-dependent. In bvFTD, reduced insight and behavioral dysregulation may undermine the adherence and reliability of self-reports. In patients with PPA, aphasia may lead to missed or delayed reporting of discomfort. For these reasons, structured monitoring with caregiver input must be routine.

With rTMS, the prevalent AEs are transient headache, scalp discomfort, and local muscle twitching. Patients less commonly reported fatigue, mood changes, or sleep disturbances. Seizures remain a rare but high-consequence risk, warranting careful screening and strict adherence to safety guidelines, particularly in individuals with a history of seizures, structural brain lesions, recent medication changes that lower the seizure threshold, or relevant comorbidities (Rossi et al., 2021). Typical AEs of tDCS/tACS include tingling, itching, erythema, warmth, and mild headache. Good electrode contact and skin care reduce irritation and the risk of skin burns; however, implanted electronic devices, scalp lesions, and recent cranial surgery require additional caution (Bikson et al., 2016; Antal et al., 2017). tPBM is usually well-tolerated, but its long-term safety in neurodegenerative diseases, especially dose–response relationships and interactions with concomitant medications, remains incompletely characterized. Therefore, eye protection, dose control, adherence checks, and structured AE checklists are required (Li et al., 2024; Lin et al., 2024). Future trials should use standardized AE frameworks, systematically capture caregiver reports, and prespecify stopping rules. Safety reporting should be harmonized across modalities to support cross-study comparisons. A summary of previous NIBS studies is provided in Table 1, and an evidence matrix by clinical subtype and stimulation modality is provided in Table 2.

**TABLE 1:** Summary of non-invasive brain stimulation studies in the frontotemporal dementia spectrum.

References	Sample	Study design	Stimulation modality	Target	Protocol	Primary outcomes
Antczak et al., 2018	bvFTD *(n* = 9), svPPA (*n* = 1), nfvPPA (*n* = 1)	Single-arm, pre–post comparison	rTMS (10 Hz)	Bilateral DLPFC	10 consecutive days	Improved MoCA scores, letter-number cancelation test scores, and Stroop test performance (shorter reading time and fewer errors); Frontal Behavioral Inventory (FBI) scores also improved.
Huey et al., 2007	bvFTD (*n* = 9), PPA (*n* = 1)	Randomized, double-blind, crossover	tDCS (2 mA)	Anode: left DLPFC; cathode: right supraorbital region	2 mA, 40 min (20 min before and 20 min after testing), single session	No significant between-condition differences in verbal fluency or changes in the Neuropsychiatric Inventory (NPI).
Agarwal et al., 2016	One female patient with bvFTD	Single case report	tDCS (2 mA)	Anode: left DLPFC; cathode: right supraorbital region	2 mA, 40 min; 5 consecutive days, twice daily	Marked improvement in behavioral disturbances and socio-occupational functioning, sustained for several months.
Ferrucci et al., 2018	bvFTD (*n* = 8), PPA (*n* = 4)	Randomized, double-blind crossover (active vs. sham)	tDCS (2 mA)	–	2 mA, 20 min/session; 5 consecutive days	tDCS significantly reduced total NPI scores with effects lasting up to 4 weeks; simple visual reaction time was shortened; EEG showed reduced low-frequency power with relative normalization of high-frequency activity.
Cotelli et al., 2018	bvFTD (*n* = 16)	Randomized, double-blind, single-session stimulation	tDCS (1.5 mA)	Medial frontal cortex (MFC; electrode at Fpz; cathode between Oz and inion)	Single session, 10 min	Significant improvement in accuracy of understanding communicative intentions in theory-of-mind (ToM) tasks.
Benussi et al., 2020a	Symptomatic FTD (*n* = 55) and presymptomatic GRN mutation carriers (*n* = 15)	Randomized, double-blind, sham-controlled	tDCS (2 mA)	Anode: left prefrontal cortex	2 mA, 20 min/session; 5 days/week for 2 weeks	In the active tDCS group, clinical ratings (behavior and executive function) and TMS-derived measures of short-interval intracortical inhibition/facilitation improved significantly; presymptomatic carriers also showed benefits in cortical physiological measures.
Sanches et al., 2022	bvFTD (*n* = 12)	Double-blind, crossover, single-session stimulation	tDCS (1.59 mA)	Anode: left DLPFC; cathode: right DLPFC; sham over left DLPFC	Single session, 1.59 mA, 20 min	No significant improvement on language-dominant outcomes (naming and semantic judgment).
Tippett et al., 2024	bvFTD (*n* = 1)	Single case, pre-post comparison	tDCS + computerized cognitive training (CCT)	Anode: left DLPFC; cathode: right DLPFC	∼10 weeks (46 sessions total)	Sustained improvements in language, attention, and functional tasks; at follow-up, the caregiver reported improved activities of daily living.
Pini et al., 2022	bvFTD (*n* = 20)	Randomized, double-blind	tDCS (2 mA)	Anode: salience network (SN); cathode: default mode network (DMN)	2 mA, 25 min/session, 5 days/week for 2 weeks	Anodal stimulation targeting the SN significantly improved language, whereas cathodal stimulation targeting the DMN significantly reduced NPI scores. Post-cathodal stimulation, behavioral symptoms improved; post-anodal stimulation, cognition improved. No significant changes were observed in functional connectivity or perfusion.
Finocchiaro et al., 2006	PPA (*n* = 1)	Single case report	rTMS	Left DLPFC	Two rTMS sessions and one sham session (pre-post assessments)	Significant and relatively sustained improvement in verb generation.
Margolis et al., 2019	PPA (*n* = 6)	Single-arm, pre-post comparison	rTMS (20 Hz)	Left or right DLPFC	Single session, 10 min	Left DLPFC rTMS may be more beneficial than right DLPFC; left DLPFC rTMS significantly improved action naming and overall cognition, with near-significant improvement in single-word reading.
Cotelli et al., 2012	PNFA (*n* = 10), SD (*n* = 4)	Randomized, double-blind, sham-controlled	rTMS (20 Hz)	Left DLPFC, right DLPFC, and sham group	Single session; short train (500 ms)	PNFA: significant improvement in action naming accuracy, with no facilitation for object naming; SD: no facilitation for either action or object naming.
Pytel et al., 2021	nfvPPA (*n* = 14), svPPA (*n* = 6)	Randomized, double-blind, crossover; individualized targeting	rTMS	Left IFG (9 nfvPPA); left superior frontal gyrus (3 nfvPPA); left DLPFC (1 nfvPPA and 5 svPPA); right superior frontal gyrus (1 nfvPPA); left anterior temporal lobe (1 svPPA)	Excitatory protocol: 20 Hz; inhibitory protocol: 1 Hz; 15 consecutive days	Improved language performance (e.g., spontaneous speech); caregiver-reported improvements (subjective change, apathy/depression); metabolic changes suggesting enhanced synaptic activity.
Huang et al., 2023	nfvPPA (*n* = 16), svPPA (*n* = 12), lvPPA (*n* = 12)	Randomized, double-blind, sham-controlled	rTMS (10 Hz)	Right-handed: left DLPFC; left-handed: right DLPFC	5 days/week for 4 weeks	Improvements in BNT and WAB were significantly greater in the active rTMS group and remained observable at 6-month follow-up; improvements in BNT, CAL, and WAB did not differ significantly across PPA variants.
Fernández-Romero et al., 2025	PPA (*n* = 63): nfvPPA (*n* = 24), svPPA (*n* = 12), lvPPA (*n* = 27)	Prospective, double-blind, parallel-group randomized clinical trial	iTBS (50 Hz) + language therapy	Left DLPFC	2 weeks, 5 days/week (10 sessions), followed by a 22-week maintenance phase (once weekly)	Slowed language and functional decline and attenuated regional metabolic deterioration.
Paranhos et al., 2025	lvPPA (*n* = 4), nfvPPA (*n* = 2), svPPA (*n* = 1), PPAOS (*n* = 3)	Double-blind, sham-controlled, crossover; individualized design	iTBS	Left caudal middle frontal gyrus	10 consecutive days	Language functions corresponding to individual weaknesses on language testing improved; functional connectivity increased within language networks across subtypes and in other cognition-related networks.
Wang et al., 2013	nfvPPA (*n* = 1)	Single case, pre-post comparison	tDCS (1.2 mA)	Morning: anode over left posterior perisylvian region (PPR); afternoon: anode over left Broca’s area	20 min/session; 5 days sham + 5 days active stimulation	Compared with sham, the active phase showed improved language performance and cortical activation.
Tsapkini et al., 2014	PPA (*n* = 6)	Controlled, crossover (tDCS + training vs. sham + training)	tDCS (1–2 mA) + language training	Left inferior frontal gyrus (IFG)	20 min/session; 5 days/week for 3 weeks	tDCS enhanced training effects, with generalization to untrained items and better maintenance.
Cotelli et al., 2014	PPA (*n* = 16)	Randomized, double-blind, sham-controlled	tDCS + language training	Left DLPFC	25 min stimulation + 25 min language training per session; 5 days/week for 2 weeks	tDCS + training yielded greater naming gains than sham + training, with more durable effects.
Gervits et al., 2016	lvPPA (*n* = 4), naPPA (*n* = 2)	Single-arm, pre-post comparison	tDCS (1.5 mA)	Anode: left frontotemporal region (F7); cathode: left occipitoparietal region (O1)	1.5 mA, 20 min/day, 10 days (2 weeks)	Improved language abilities in speech production and grammatical comprehension.
Tsapkini et al., 2018	nfvPPA (*n* = 14), lvPPA (*n* = 12), svPPA (*n* = 10)	Randomized, double-blind, sham-controlled crossover	tDCS (2 mA) + language training	Anode: left frontal site (F7); cathode: right cheek	2 mA, 20 min; 5 days/week for 3 weeks	tDCS enhanced language therapy effects (particularly naming/writing measures), but responses differed by variant; no significant benefit was observed in the svPPA group.
Roncero et al., 2019	nfvPPA (*n* = 4), lvPPA (*n* = 4), svPPA (*n* = 4)	Randomized, double-blind crossover (active vs. sham)	tDCS (2 mA) + naming training	Anode: left inferior parietotemporal region (P3) or left DLPFC (F3)	2 mA, 30 min/session, 10 sessions total	tDCS enhanced language therapy effects; at follow-up, the temporoparietal stimulation group showed the greatest improvement. Among temporoparietal-stimulated patients, svPPA exhibited the smallest naming improvement for untrained items after stimulation.
Dugas et al., 2025	PPA (*n* = 8)	Randomized, double-blind	HD-tDCS (1 mA)	Anode: left IFG (LIFG) or pre-supplementary motor area (pre-SMA)	1 mA, 20 min; 5 days/week for 2 weeks	The left IFG group showed significant improvement in letter fluency; in both groups, some patients achieved clinically meaningful improvement; ERP modulation was also observed.
de Aguiar et al., 2020	nfvPPA (*n* = 15), lvPPA (*n* = 17), svPPA (*n* = 8)	Randomized, double-blind, sham-controlled	tDCS (2 mA) + language training	Anode: left IFG (10–20 system F7); cathode: right cheek	20 min stimulation + 25 min training per session; 5 days/week for 3 weeks	Baseline cognitive and language performance predicted additional tDCS gain; language improvement remained significant at 2-month follow-up in the active condition.
de Aguiar et al., 2020	nfvPPA (*n* = 9), lvPPA (*n* = 14), svPPA (*n* = 7)	Randomized, double-blind, sham-controlled crossover	tDCS (2 mA) + language training	Anode: left IFG (10–20 system F7); cathode: right cheek	20 min stimulation + 25 min training per session; 5 days/week for 2–3 weeks	Regional brain volume/atrophy predicted incremental benefit from tDCS. For trained words, volumes of the left angular gyrus and left posterior cingulate cortex predicted additional tDCS gain; for untrained words, volumes of the left middle frontal gyrus, left supramarginal gyrus, and right posterior cingulate cortex predicted additional tDCS gain.
McConathey et al., 2017	PPA (*n* = 15)	Randomized, double-blind, sham-controlled	tDCS (1.5 mA)	Anode: left prefrontal region (10–20 system F7)	1.5 mA, 20 min; 5 days/week for 2 weeks	Baseline language severity influenced treatment response.
Borrego-Écija et al., 2023	nfvPPA (*n* = 6), lvPPA (*n* = 5), svPPA (*n* = 4)	Double-blind, randomized, crossover, sham-controlled	Multifocal/ multichannel tDCS (2 mA) + language training	Anodes: C1, F7, FC1, FC5, Fpz, P7, PO8	2 mA, 26 min; 5 days/week for 2 weeks	Multifocal tDCS was not superior to speech-language therapy alone.

PPA, primary progressive aphasia; tDCS, transcranial direct current stimulation; rTMS, repetitive transcranial magnetic stimulation; iTBS, intermittent theta-burst stimulation; DLPFC, dorsolateral prefrontal cortex; IFG, inferior frontal gyrus; nfvPPA, non-fluent/agrammatic variant primary progressive aphasia; PNFA, progressive non-fluent aphasia; svPPA, semantic variant primary progressive aphasia; lvPPA, logopenic variant primary progressive aphasia.

**TABLE 2:** Evidence matrix of non-invasive brain stimulation by clinical subtype and modality.

Clinical subtype	rTMS/TBS	tDCS	tACS	tPBM
bvFTD	Limited; small uncontrolled signals	Moderate; mixed controlled evidence; behavior and executive signals	Very limited; no clear subtype trials	Very limited; no robust clinical data
nfvPPA	Moderate; language/naming improvements	Moderate; often with language therapy; variable	Very limited	Very limited
svPPA	Limited-moderate; variable benefit	Limited; smaller gains; limited generalization	Very limited	Very limited
lvPPA	Moderate; small trials; language signals	Moderate; training-augmented effects	Very limited	Very limited
Genetic/at-risk (e.g., GRN)	Very limited	Limited; early physiological/clinical signals	Very limited	Very limited

Evidence categories are qualitative and reflect study design, sample size, and consistency across outcomes.

## 3 Conclusion

As summarized in Table 2, the most developed evidence in the FTD spectrum currently centers on tDCS and rTMS/iTBS in PPA variants, whereas tACS and tPBM remain sparsely studied.

### 3.1 Limitations of the current evidence base

The current NIBS literature in FTD is limited by small sample sizes and substantial heterogeneity. Most available studies are small, often exploratory, and variably controlled. At least four major sources of variation recur across the literature: clinical phenotype (e.g., bvFTD, nfvPPA, and svPPA); disease stage and regional atrophy burden; stimulation design (including target, targeting method, montage, intensity, frequency, number of sessions, and maintenance schedule); and outcome selection, including whether stimulation is combined with speech-language or cognitive therapy. These factors are likely to interact and may help explain why broadly similar protocols yield different results across cohorts. For example, a left frontal montage may be appropriate for lexical retrieval in nfvPPA but less effective in advanced bvFTD or svPPA, where atrophy, network disconnection, or mismatch between the stimulation target and the dominant clinical deficit may reduce effective target engagement. Accordingly, negative or null findings should be considered informative rather than merely inconclusive, as they may reflect insufficient dose, single-session exposure, suboptimal targeting, advanced disease stage, or insensitive endpoints rather than true lack of therapeutic potential. Outcome selection remains an additional limitation. Several studies have focused on short-term task performance, whereas clinically meaningful endpoints–such as behavior, functional communication, activities of daily living, and caregiver burden–have not been consistently prespecified or reported. Follow-up durations are typically short, leaving durability and dose–response relationships uncertain, and physiological target engagement is rarely demonstrated. Without biomarkers of network integrity or atrophy, together with physiological readouts, it remains difficult to distinguish true lack of efficacy from inadequate engagement of the intended circuit. In this context, mechanistic hypotheses exceed the strength of the available clinical evidence and should be interpreted as supportive frameworks for future translational research rather than validated explanations of treatment response. Progress in the field will therefore depend on preregistered, sham-controlled, adequately powered trials with syndrome-stratified recruitment, harmonized core outcomes, transparent reporting of null results, and longer follow-up.

### 3.2 Translational priorities and future directions

Non-invasive brain stimulation remains an early-stage intervention in FTD; however, it offers a testable route to symptom-oriented and potential mechanism-informed care. The next priority is adequately powered, sham-controlled trials that stratify patients by clinical syndrome (bvFTD, nfvPPA, and svPPA) and, where feasible, by biological features such as atrophy patterns, network connectivity, genotype, and sex. Such stratifications are not simply a methodological refinement; in a disorder defined by selective network vulnerability, they are essential to reduce response variance and to identify subgroup-specific effects. Target selection should be guided by multimodal imaging and neurophysiology. Mapping syndrome-specific network disruptions can support individualized localization and parameter selection. In parallel, trials should incorporate prespecified biomarkers of target engagement–connectivity metrics, neurophysiological measures, or dose–field estimates in the context of atrophy, such that clinical outcomes can be interpreted mechanistically rather than descriptively. The second priority is durability. Most studies used short courses and brief follow-ups. Future protocols should explicitly test maintenance strategies and include follow-ups long enough to assess persistence and dose–response. When behavior or functional communication is the primary clinical objective, outcome measures should reflect real-world impact, including caregiver-reported assessments. Future studies should also report null findings transparently and adopt harmonized core outcome sets that extend beyond task performance to include behavior, functional communication, activities of daily living, and caregiver-relevant outcomes. Mechanistic investigations should move beyond general descriptions of “network modulation” and instead assess whether NIBS can engage FTD-relevant pathways with measurable biological signatures. Although emerging neuromodulation studies may help inform this translational framework, the supporting evidence in FTD remains limited and largely indirect. For example, deep brain stimulation has been reported to enhance synaptic activity and promote TFEB nuclear translocation in primary neurons derived from FTD transgenic mice, thereby facilitating the clearance of MAPT/tau oligomers (Akwa et al., 2023). Similarly, altered activity of GABAergic interneurons has been described in AD and FTD models, and optogenetically driven 40-Hz stimulation has been shown to restore gamma oscillations, increase microglial activation, and reduce amyloid-β and phosphorylated tau in AD models, suggesting potential relevance of rhythm-based interventions to tau-related pathology in FTD (Assogna et al., 2021). Nevertheless, these observations should be interpreted as indirect and hypothesis-generating, rather than as established explanations of clinical benefit in FTD.

### 3.3 Expert opinion

#### 3.3.1 Expert commentary

Frontotemporal dementia lacks effective disease-modifying therapies, and clinical phenotypes reflect distinct network vulnerabilities. Across rTMS/TBS, tDCS/tACS, and tPBM, published studies suggest that NIBS can be delivered safely and may yield symptom-linked signals in selected domains (particularly language outcomes in PPA). However, evidence is limited by small samples, heterogeneous protocols, and incomplete sham control. The evidence matrix in this review highlights where signals cluster and where data are absent, providing a practical guide for syndrome-stratified protocol selection and outcome planning.

#### 3.3.2 Five-year view

Progress is likely to come from coordinated, multicenter, sham-controlled trials that pre-specify clinically meaningful endpoints and incorporate target-engagement measures. Imaging-informed personalization (atrophy-aware neuronavigation, network-based targets) and combination strategies (stimulation paired with language/cognitive training) should improve effect sizes and generalization. Home-based, monitored tES and tPBM may expand dose and maintenance paradigms, whereas digital biomarkers and caregiver-reported outcomes can improve sensitivity to change. Ultimately, NIBS is most plausibly positioned as a personalized, syndrome-specific adjunct rather than a one-size-fits-all intervention.
